# Supplementary material for: Deciphering the role of accessory proteins in Arabidopsis chloroplast editosomes via interaction with a synthetic PPR-PLS factor in E. coli
Source: Nucleic Acids Res. 2025 Jun 11;53(11):gkaf483. doi: 10.1093/nar/gkaf483 (PMC12153344; doi:10.1093/nar/gkaf483)
Supplement: gkaf483_Supplemental_File [file gkaf483_supplemental_file.pdf]

Table S1. Primers used in this study

goal	gene	Forward	Reverse	Comments
clone in pETDuet	dsn3PLS-DYW			genescript. The dsn3PLS-DYW delivered cloned into NcoI
				and BamHI of pETDuet
		BamHI-rpoA-C200-F1	SalI-rpoA-C200-R1	to clone the target into petDuet-dsn3PLS-DYW
target	*rpoA*	GATCaaatagaaggaacatgtattacacgtgcaaaatctgaga	TCGAtctcagattttgcacgtgtaatacatgttccttctattt	
		EcoRI-RIP2-F1	SacI-RIP2-R1	to express RIP2 with a N-His tag
RIP2 in MCS1	*RIP2*	gaattccGGAGCTAACCGGTCAGG	gagctcTCATCTTGTGTTTTCTCTGC	
		NdeI-RIP2-F1	BglII-RIP2-R1	to express RIP2 with a C-S-tag
RIP2 in MCS2	*RIP2*	catatgGGAGCTAACCGGTCAGG	agatctaaTCTTGTGTTTTCTCTGCGGC	
		Q5-RIP2+Stop-F1	Q5-RIP2-R1	use these primers with Q5 site directed mutagenesis on
RIP2 without tag	*RIP2*	TAAttaGATCTCAATTGGATATCGGCC	TCTTGTGTTTTCTCTGCGGC	pCDF-RIP2 in MCS2 to add a stop codon and remove the S tag
		BamHI-RIP9-F1	EcoRI-RIP9-R1	to express RIP9 with a N-His-tag
RIP9 in MCS1	*RIP9*	ggatcccGCGACGGTGGATTCG	gaattcTTAAGAGGAATCAGAGGCTG	
		Nde-RIP9-F1	BglII-RIP9-R1	to clone RIP9 in MCS2 (+ stop)
RIP9 in MCS2	*RIP9*	catatgGCGACGGTGGATTCG	agatctTTAAGAGGAATCAGAGGCTG	
		Q5-RIP9-S-tag-F1	Q5-RIP9-S-tag-R11	use these primers with Q5 site directed mutagenesis on pCDF-RIP9
RIP9 with a S-tag	*RIP9*	AAAGAAACCGCTGCTGCG	AGAGGAATCAGAGGCTGCTG	in MCS2 to remove the stop codon and have the S tag in frame with RIP9
		BamHI-ORRM1-F1	EcoRI-ORRM1-R1	to express ORRM1 with a N-His-tag
ORRM1 in MCS1	*ORRM1*	ggatcccTCATCTTCTGCAATTTCCGC	gaattcCTAGAGCCCGAAACTTGG	
		NdeI-ORRM1-F1	XhoI-ORRM1-R1	to express ORRM1 with a C-S-tag
ORRM1 in MCS2	*ORRM1*	catatgTCATCTTCTGCAATTTCCGC	ctcgagGAGCCCGAAACTTGGTTG	
			XhoI-ORRM1+ stop-R1	to express ORRM1 without a tag
			ctcgagtcaGAGCCCGAAACTTGGTTG	
		BamHI-OZ1-F1	SacI-OZ1-R1	to express OZ1 with a N-His-tag
OZ1 in MCS1	*OZ1*	ggatccgGCCGCAGATTTTGTTCC	gagctcTCATTTATCTCCTTTACCAGTGG	
		NdeI-OZ1-F1	BglII-OZ1+stop-R1	to express OZ1 without a tag
OZ1 in MCS2	*OZ1*	catatgGCCGCAGATTTTGTTCC	agatctTCATTTATCTCCTTTACCAGTGG	
		Q5-OZ1-S-tag-F1	Q5-OZ1-S-tag-R11	use these primers with Q5 site directed mutagenesis on pCDF-OZ1 in
OZ1 with a S-tag	*OZ1*	AAAGAAACCGCTGCTGCG	TTTATCTCCTTTACCAGTGGGATC	MCS2 to remove the stop codon and have the S tag in frame with OZ1
		BamHI-ISE2-F1	SacI-ISE2-R1	to express ISE2 with a N-His-tag
ISE2 in MCS1	*ISE2*	ggatcccCAGCTCTACGAGGAAGA	gagctcTTAACCGGCAAGCTCA	
		BglII-ISE2-F1	AatII-ISE2-R1	to express ISE2 with a C-S-tag
ISE2 with a S-tag	*ISE2*	agatctCCAGCTCTACGAGGAAGA	gacgtcACCGGCAAGCTCACT	
			AatII-ISE2+stop-R1	to express ISE2 without a tag
			gacgtcTTAACCGGCAAGCTCACT	
		SalI-rpoA-C200-F1	NotI-rpoA-C200-R1	to clone rpoA in the downstream of MCS1 and have restriction sites
	rpoA	TCGAaaatagaaggaacatgtattacacgtgcaaaatctgaga	GGCCtctcagattttgcacgtgtaatacatgttccttctattt	upstream to be able to clone dsn3PLS-DYW
		BamHI-dsn3PLS-F1	PstI-dsn3PLS-DYW-R1	to clone dsn3PLS-DYW with a N-His-tag in pETDuet-rpoA
dsn3PLS in MCS1	*dsn3PLS-DYW*	ggatccaATGGGAAATTCTGTTTTTTCTTGG	ctgcagttaCCAATAATCTCCACAAGAAC	
		NdeI-dsn3PLS-F1	KpnI-dsn3PLS-R1	to clone dsn3PLS-DYW with aC-S-tag in pETDuet-rpoA
dsn3PLS in MCS2	*dsn3PLS-DYW*	catatgATGGGAAATTCTGTTTTTTCTTGG	ggtaccCCAATAATCTCCACAAGAACAAG	
		Q5+4His-F1	Q5+4His-R1	to increase the His tag in Duet vector from 6xHis to 10xHis
increase His tag		CATCACCATCATCATCACCATCATCACCACAGC	GCTGCTGCCCATGGTATATC	
		rpoA_REMSA		to use in gel shift assays and visualize the bound and unbound RNA target
REMSA experiments	*rpoA*	/5Cy5/AUGUAUUACACGUGCAAAAUCUGAGA		
		dsn3PLS-DYW-F1	T7 Terminator Primer	to perform RT-PCR in order to quantify the level of editing extent of the
RT-PCR	*rpoA*	GGAAGATGGGATGATGTTGCT	GCTAGTTATTGCTCAGCGG	rpoA-C200 target by bulk-sequencing
		BamHI-CLB19-F1	SalI-CLB19-R1	
CLB19 in MCS1	*CLB19*	ggatccGAAGGAATTCTCCGATATGACAC	gtcgacTCAAGCATTGAGGAGATCACC	to clone CLB19 in the MCS1 of petDuet-1
		SalI-ORRM1-F1	BamHI-ORRM1-R1	
ORRM1 in pMAL-c6T	*ORRM1*	gtcgacTCATCTTCTGCAATTTCCGCA	ggatccCTAGAGCCCGAAACTTGGTT	to clone ORRM1 in the pMAL-c6T vector (with a MBP tag at its N terminus)
		BamHI-MBP-ORRM1-F1	EcorRI-MBP-ORRM1-R1	
MBP-ORRM1 in MCS1	*MBP-ORRM1*	ggatcccGAAGAAGGTAAACTGGTAATCTGG	gaatccCTAGAGCCCGAAACTTGGTTG	to clone MBP-ORRM1 in the MCS1 of pCDF after amplifying it from pMAL-c6T

Table S2 . Code of the bacterial samples assayed

sample#		dsn-3PLSDYW + rpoA	accessory proteins
1		YES	pCDF-HisRIP2
2		YES	pCDF-HisRIP9
3		YES	pCDF-HisORRM1
4		YES	pCOLA-HisRIP2
5		YES	pCOLA-HisRIP9
6		YES	pCDF-HisORRM1 + pCOLA-HisRIP2
7		YES	pCDF-HisORRM1 + pCOLA-HisRIP9
8		YES	pCDF-OZ1 + pCOLA-HisRIP2
9		YES	pCDF-ISE2 + pCOLA-HisRIP2
10		YES	pCDF-ISE2 + pCOLA-HisORRM1
11		YES	pCOLA-HisORRM1
12	+IPTG		
13	-IPTG		
14		YES	

Table S3 . Annotation of the coding sequences where off-target editing occurs and change of amino acids caused by these editing events

site[a]	strand	strand_annotation	gbkey	gene_id	gene_biotype	gene	protein_id	product	AA change	AA#	editing extent %[b]
301209	+								NA		15
433327	-	+	CDS	SR36_02065	protein_coding		AJH09347.1	hypothetical protein	NA		14
779169	-	-	CDS	SR36_03890	protein_coding		AJH09667.1	membrane protein	GLN>STOP	33	11
846395	+	-	CDS	SR36_04215	protein_coding		AJH09725.1	hypothetical protein	NA		1
957341	+	+	CDS	SR36_04730	protein_coding		AJH13067.1	hypothetical protein	PHE>PHE	4	1
998460	-	-	CDS	SR36_04915	protein_coding		AJH09852.1	hypothetical protein	SER>PHE	48	6
1115962	-	-	CDS	SR36_05555	protein_coding	rne	AJH09969.1	ribonuclease E	ILE>ILE	46	1
1610233	-	-	CDS	SR36_08120	protein_coding		AJH10433.1	beta-D-glucuronidase	VAL>VAL	67	15
1650242	+	+	CDS	SR36_08325	protein_coding		AJH10471.1	superoxide dismutase	ARG>ARG	168	1
1724437	+								NA		13
1821466	+	-	CDS	SR36_09215	protein_coding	rrmA	AJH10634.1	23S rRNA methyltransferase	NA		26
1838803	-	-	CDS	SR36_09320	protein_coding		AJH10652.1	membrane protein	LEU>LEU	226	10
1941416	+								NA		21
1967867	-	-	CDS	SR36_09990	protein_coding		AJH10764.1	mannose-1-phosphate guanylyltransferase	PRO>LEU	452	17
2437684	-	+	Gene	SR36_12140	pseudogene	hycD			NA		12
2469966	+	-	CDS	SR36_12285	protein_coding		AJH11199.1	GTP-binding protein Der	NA		6
2728082	+	+	CDS	SR36_13570	protein_coding		AJH11430.1	LOG family protein	PRO>LEU	7	3
2728396	+	+	CDS	SR36_13570	protein_coding		AJH11430.1	LOG family protein	HIS>STOP	112	1
2767599	-	-	CDS	SR36_13735	protein_coding	recC	AJH11459.1	exonuclease V subunit gamma	HIS>STOP	759	11
3018155	-	+	CDS	SR36_14940	protein_coding		AJH11685.1	hypothetical protein	NA		7
3146210	-	-	CDS	SR36_15575	protein_coding		AJH11802.1	lipoprotein NlpI	PRO>SER	127	1
3248710	+								NA		13
3406026	-								NA		2
3960072	-	-	CDS	SR36_19600	protein_coding		AJH12538.1	formate dehydrogenase	TYR>TYR	289	5
4036064	+	+	CDS	SR36_19965	protein_coding		AJH12607.1	transcriptional regulator	ARG>CYS	37	1
4058658	+	+	CDS	SR36_20095	protein_coding	rpoB	AJH12623.1	DNA-directed RNA polymerase subunit beta	SER>SER	66	1
4118470	+	-	CDS	SR36_20335	protein_coding	xylE	AJH12662.1	D-xylose transporter XylE	NA		10
4145999	+	+	CDS	SR36_20470	protein_coding	aphA	AJH12686.1	acid phosphatase	ILE>ILE	4	1
4148094	-	-	CDS	SR36_20485	protein_coding		AJH12689.1	excinuclease ABC subunit A	THR>THR	784	6
4258541	+	-	CDS	SR36_21000	protein_coding		AJH12784.1	fumarate reductase	NA		37
4289975	+	+	CDS	SR36_21155	protein_coding		AJH12811.1	isovaleryl-CoA dehydrogenase	HIS>TYR	6	16
4350628	+	+	CDS	SR36_21440	protein_coding		AJH12867.1	magnesium ABC transporter ATPase	GLN>STOP	733	1
4471230	-	-	CDS	SR36_22100	protein_coding		AJH12985.1	4-hydroxyphenylacetate permease	PRO>LEU	448	21
4495686	+	+	CDS	SR36_22230	protein_coding		AJH13005.1	DNA polymerase III subunit psi	PRO>LEU	115	5

[a]off-target editing sites are denominated by the coordinate on the bacterial chromosome

[b]the editing extent is the average of all the samples in which the off-target was detected

Table S4 . Depth coverage of the RNA-seq analysis for the bacterial samples assayed in this study

Sample	Number of Reads	Mapped Reads
1	149,347,592	128,800,565
2	162,042,216	130,075,039
3	153,377,948	107,058,018
4	88,608,512	77,681,690
5	109,602,299	93,866,143
6	140,405,427	103,518,690
7	144,883,642	108,811,156
8	111,881,087	73,554,936
9	96,387,685	68,844,679
10	107,190,467	76,295,241
11	119,315,975	102,616,950
12	59,345,539	57,979,066
13	50,914,777	50,477,615
14	134,129,709	131,873,158
MIN		50,477,615
MAX		131,873,158
AVERAGE		93,675,210

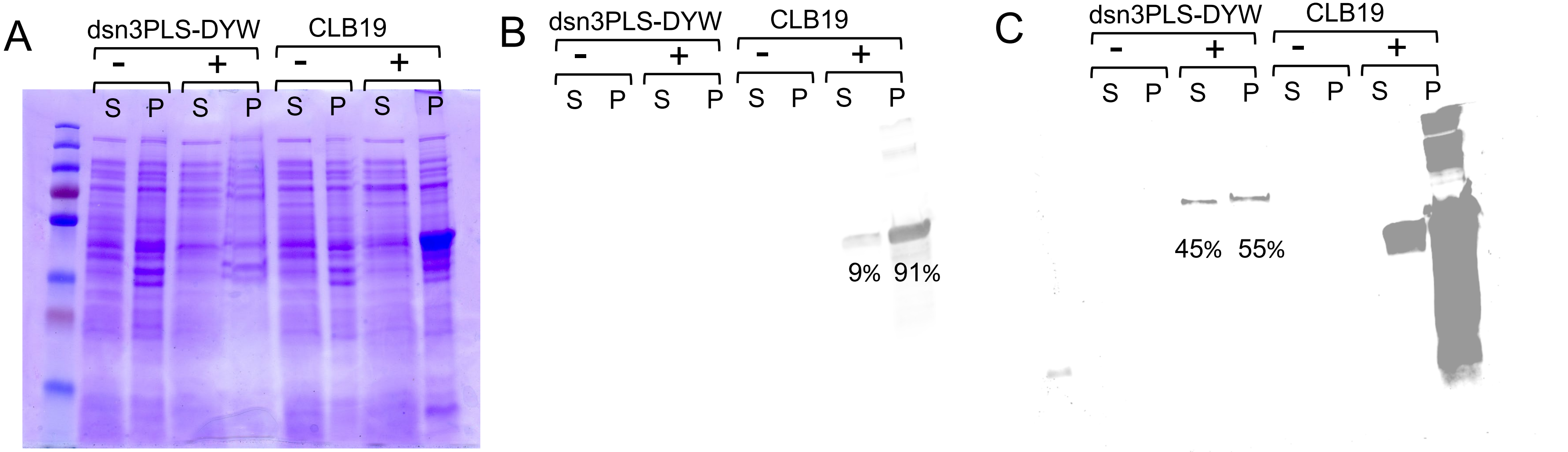

Figure S1. The natural PPR protein CLB19 is much less soluble than the synthetic PPR protein dsn3PLS-DYW. (A) Coomassie gel of bacterial protein extracts from strains expressing dsn3PLS-DYW or CLB19. -: not induced, +: induced by IPTG, S: soluble fraction, P: pellet (insoluble fraction). (B) Western blot of the gel shown in (A) with antibody against His tag, underexposed, the percentages of the protein were quantified using the ImageJ software. (C) Same western blot overexposed alowing the detection of the dsn3PLS-DYW protein.

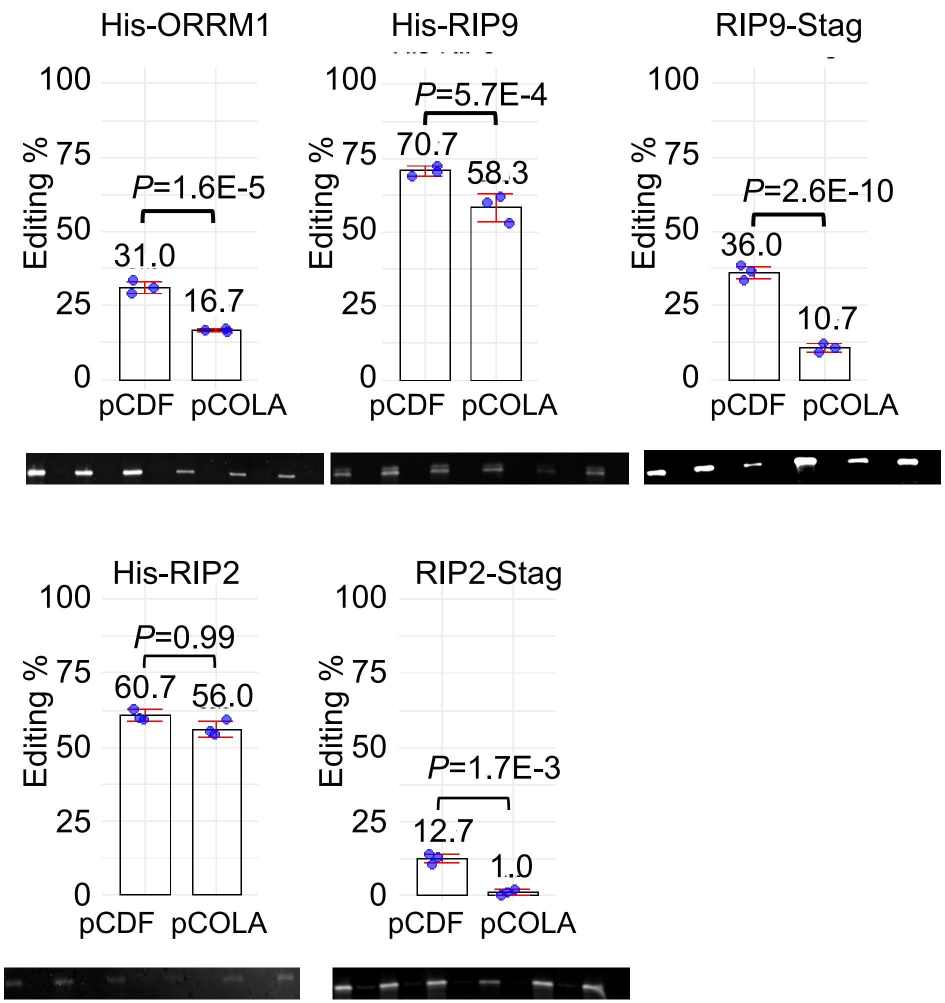

Figure S2. The expression vector used for the accessory protein can have an effect on the editing extent of the *rpoA*-C200 target. The editing extent of *rpoA*-C200 is given for three biological replicates. Below the graphs are images of the Western blots reflecting the expression of the accessory proteins with antibodies against the His tag or the S tag. The level of expression is generally lower in the pCOLA vector and the lower editing extent.

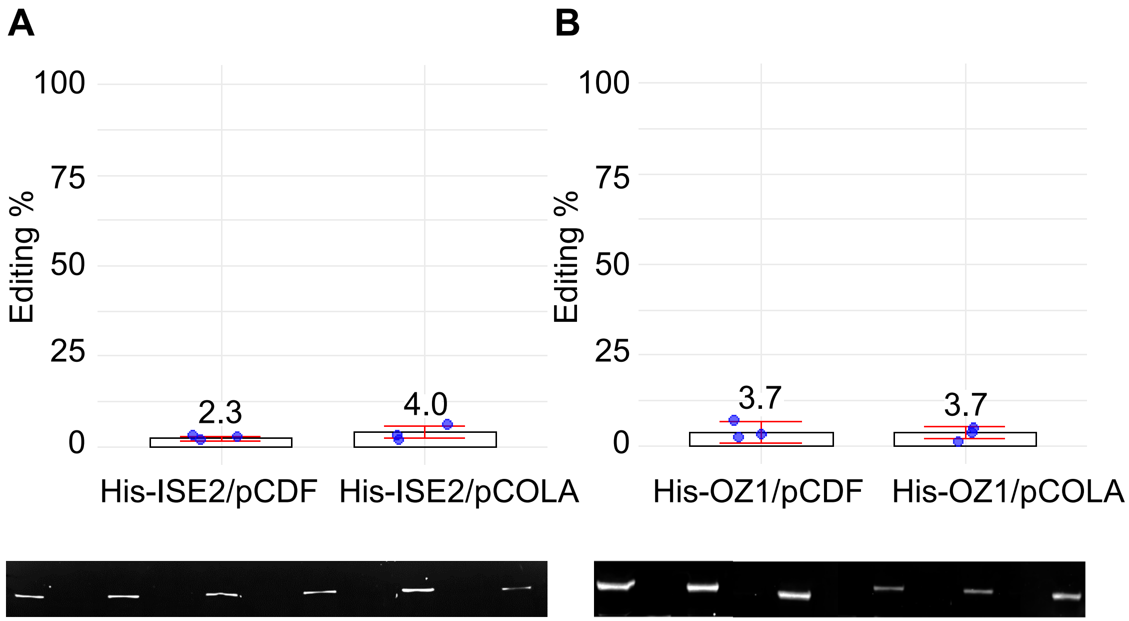

Figure S3. **OZ1 and ISE2 do not have an effect on the editing extent of the *rpoA*-C200 target when co-expressed with the synthetic factor dsn3PLS-DYW.** The graphs represent the editing extent of the *rpoA*-C200 target when dsn3PLS-DYW is co-expressed with ISE2 (A) or OZ1 (B). The editing extent of *rpoA*-C200 is given for three biological replicates. Below the graph are images of the Western blots showing that the OZ1 and ISE2 proteins are expressed.

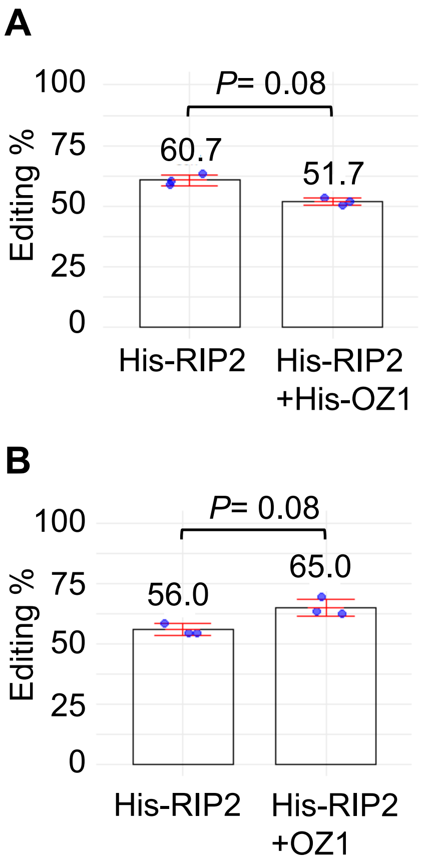

Figure S4. OZ1 might impact the effect on the editing extent of the *rpoA*-C200 target when co-expressed with the synthetic factor dsn3PLS-DYW and RIP2. The graphs represent the editing extent of the *rpoA*-C200 target when dsn3PLS-DYW is co-expressed with pCDF-HisRIP2 and pCOLA-His-OZ1(A) or pCOLA-His-RIP2 and pCDF-OZ1 (B). The editing extent of *rpoA*-C200 is given for three biological replicates.

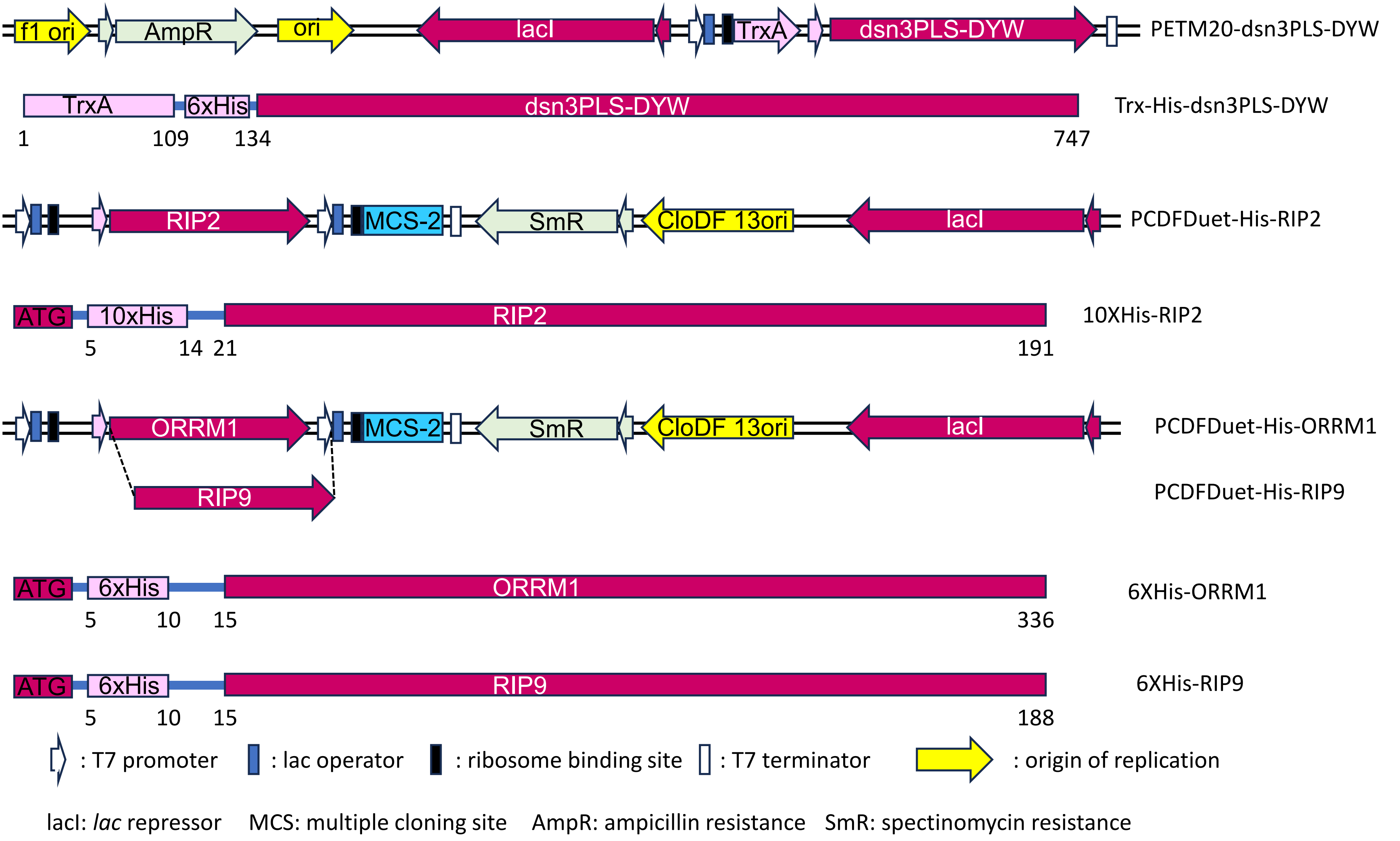

Figure S5. Vectors used to express dsn3PLS-DYW, RIP2, ORRM1, and RIP9 for their purification. Below each vector is a representation of the protein expressed. Below the protein model are the aa positions. PETM20 is an expression vector that adds a thioredoxin (TrxA) at the N-terminus of the protein to increase its solubility. RIP2, ORRM1 and RIP9 were purified by expressing them with the same pCDFDuet vector used in the bacterial experiment. We increased the 6xHis tag to a 10xHis tag in the pCDFDuet vector used to purify RIP2 to improve its purification.

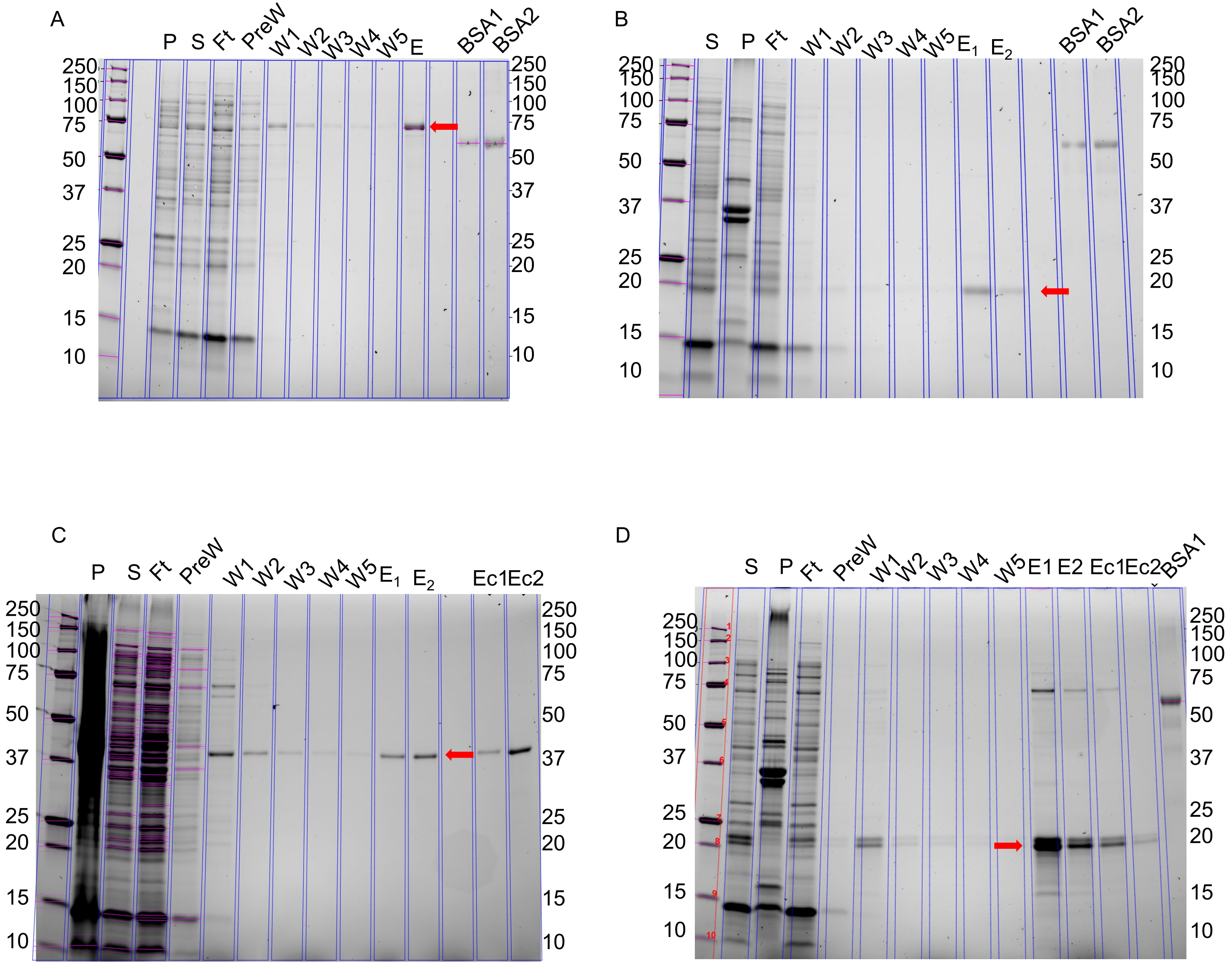

Figure S6. Purification of dsn3PLS-DYW (A), RIP2 (B), ORRM1 (C) and RIP9 (D) after induction and culture in bacteria. SDS-PAGE of the different fractions obtained during the purification of the proteins. P: total protein, S; supernatant, Ft: flow through, PreW: prewash, W1-W5: serial washes, E: elution, Ec: elution concentrated, BSA1, BSA2: BSA controls. Purified proteins are indicated by a red arrow, around 75 kDa for dsn3PLS-DYW, 20 kDa for RIP2, 37 kDa for ORRM1 and 21.5 kDa for RIP9.

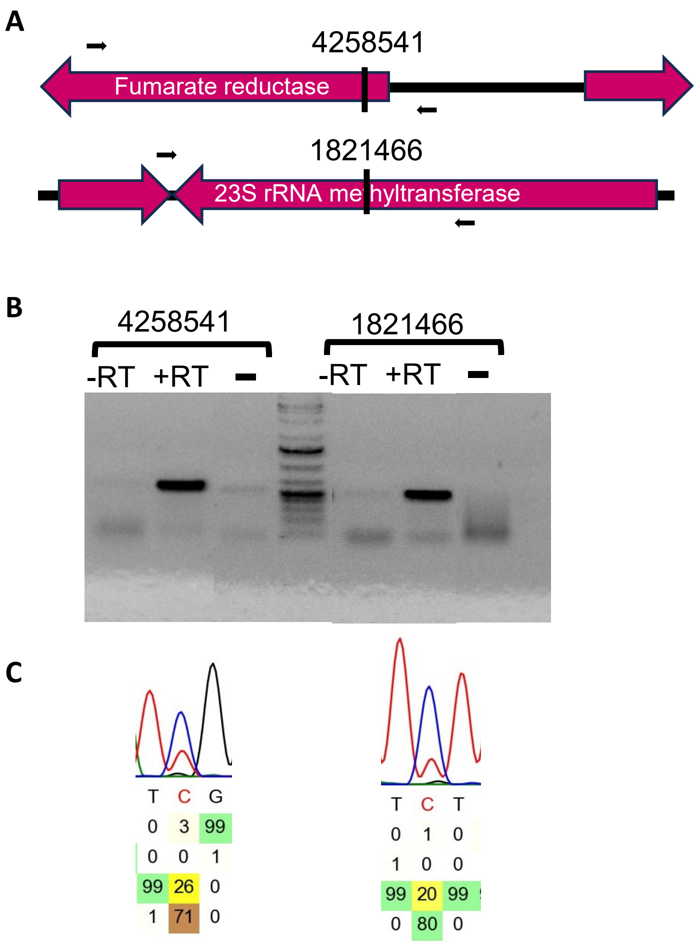

Figure S7. Bulk sequencing of RT-PCR products confirms the editing of the two highest edited off-targets, 4258541 and 1821466. (A) gene models of the coding sequences where the off targets occur. Both off-targets are on the opposite strand (+) from the annotated one (-). The arrow shoe the postion of the primers used for the RT-PCR reaction. (B) Electrophoresis gel of the RT-PCR products with their control, -RT: without reverse transcriptase, +RT: with reverse transcriptase, -: no template. The template for 4258541 is sample1 (dsn3PLS-DYW + pCDF-RIP2), the template for 1821466 is sample 2 (dsn3PLS-DYW + pCDF-RIP9). (C) Electrophoretogram of RT-PCR products with the C target in the middle of the electrophoretogram showing 3 nucleotides T C/T G (4258541) or T C/T T (1821466). Below the targeted C is the percentage of T (upper number) versus C (lower number) as computed by the BEAT software. Editing extent of 4258541 is 26% in sample1 while editing extent of 1821466 is 20% in sample 2.

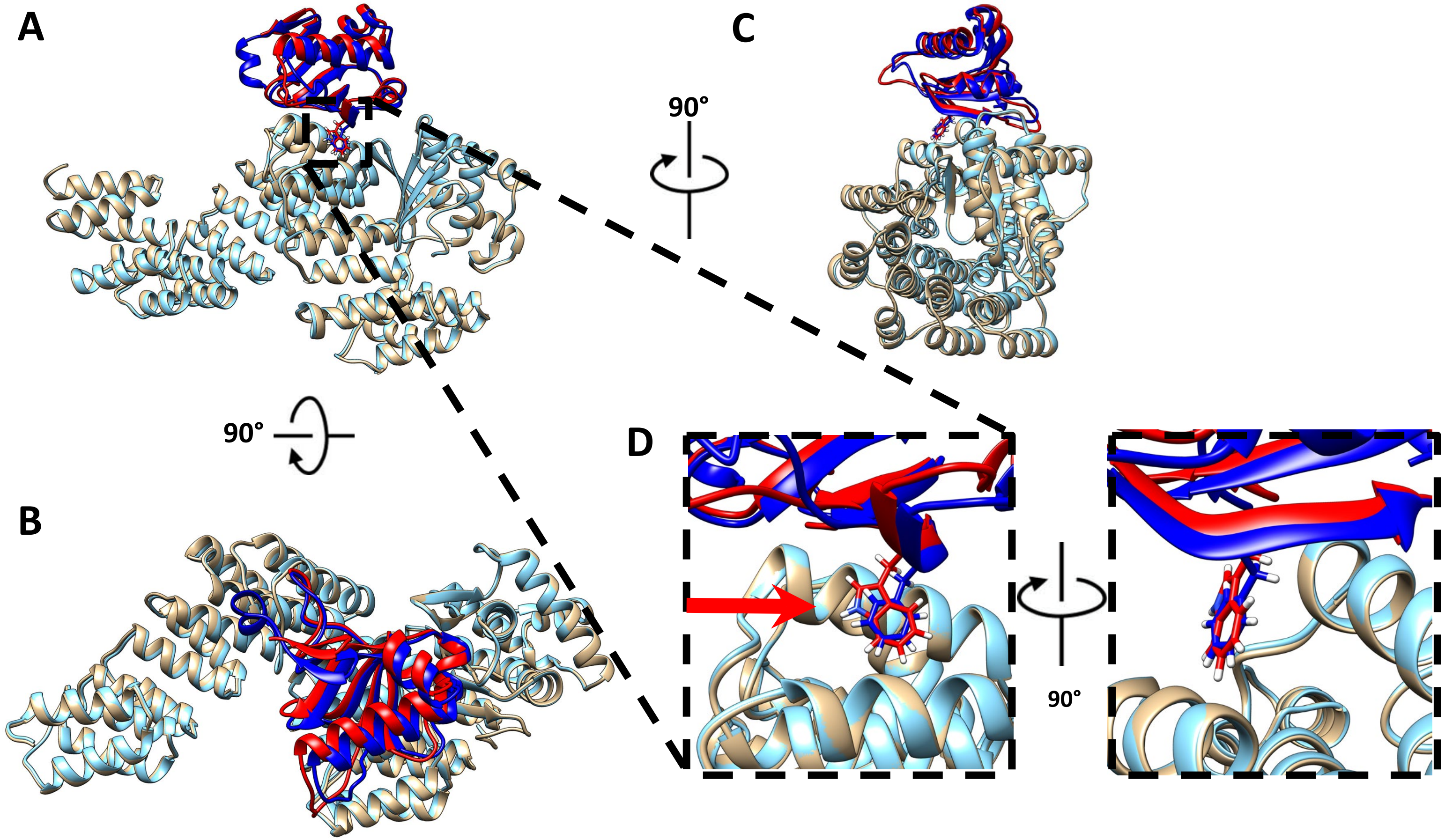

Figure S8. AlphaFold2-multimer predictions of RIP-dsn3PLS-DYW, superimposed, with each chain colour-coded. dsn3PLS-DYW (cyan), RIP2 (89-186) (blue), and RIP9 (86-192) (red). Three different views of the structures are shown, from side (**A**), top (**B**) and front (**C**). (**D**) Magnification depicting the RIP-PPR(L) predicted interaction. The arrow in panel D indicates the differential residue in RIP2 – Phe157 and in RIP9 – Trp160.

(A) dsn3PLS-DYW + RIP2(89-186) **PAE plot**

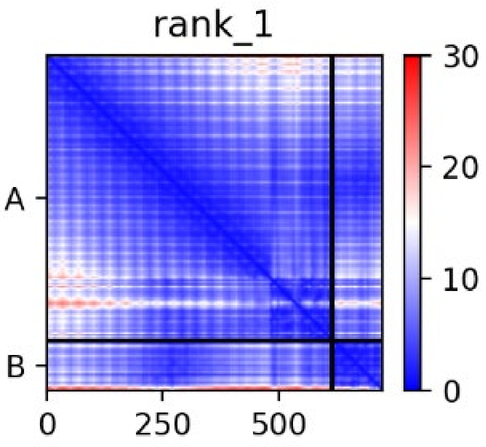

(B) dsn3PLS-DYW + RIP9(86-192) **PAE plot**

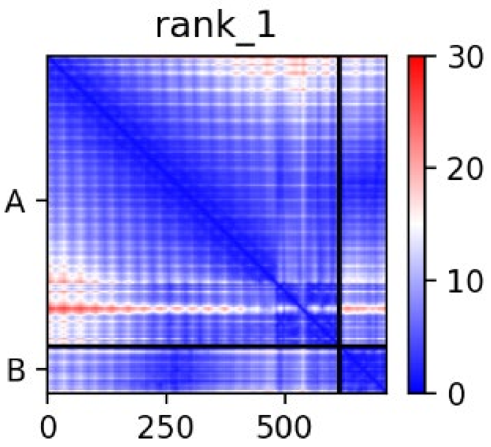

Figure S9. Predicted-Aligned Error (PAE) plots of (A) RIP2 (89-186) and (B) RIP9 (86-192) in complex with dsn3PLS-DYW.

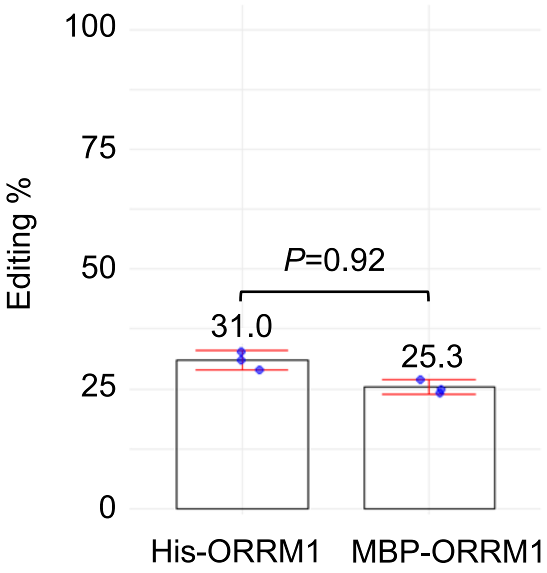

Figure S10. The substitution of His tag by MBP tag does not impact the ability of ORRM1 to allow the editing of the *rpoA*-C200 target by dsn3PLS-DYW in *E. coli*. The graphs represent the editing extent of the *rpoA*-C200 target when dsn3PLS-DYW is co-expressed with pCDF-HisORRM1(left) or pCDF-MBP-ORRM1 (right). The editing extent of *rpoA*-C200 is given for three biological replicates.

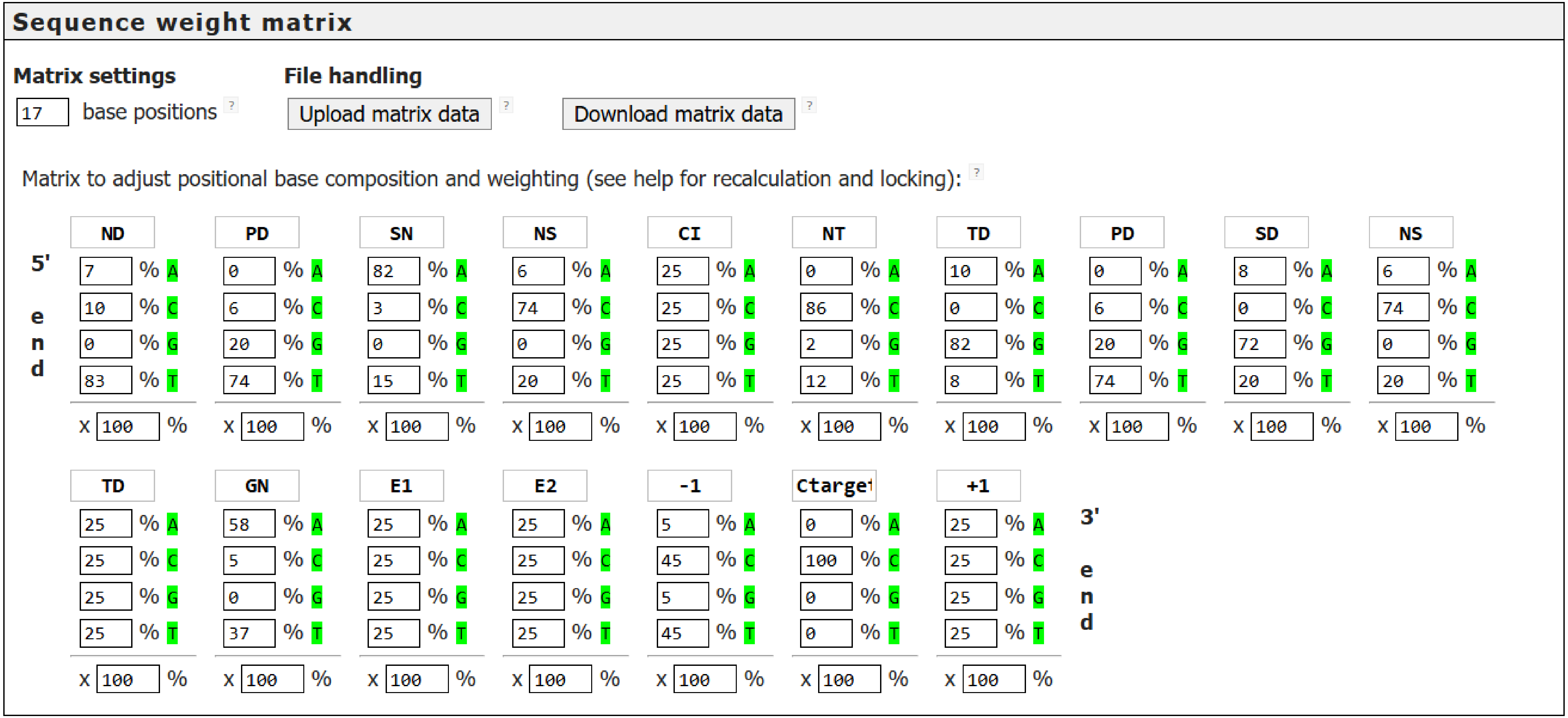
Sequence weight matrix
Matrix settings
17 base positions
File handling
Upload matrix data
Download matrix data
Matrix to adjust positional base composition and weighting (see help for recalculation and locking):
5' end
ND: 7 % A, 10 % C, 0 % G, 83 % T, x 100 %
PD: 0 % A, 6 % C, 20 % G, 74 % T, x 100 %
SN: 82 % A, 3 % C, 0 % G, 15 % T, x 100 %
NS: 6 % A, 74 % C, 0 % G, 20 % T, x 100 %
CI: 25 % A, 25 % C, 25 % G, 25 % T, x 100 %
NT: 0 % A, 86 % C, 2 % G, 12 % T, x 100 %
TD: 10 % A, 0 % C, 82 % G, 8 % T, x 100 %
PD: 0 % A, 6 % C, 20 % G, 74 % T, x 100 %
SD: 8 % A, 0 % C, 72 % G, 20 % T, x 100 %
NS: 6 % A, 74 % C, 0 % G, 20 % T, x 100 %
TD: 25 % A, 25 % C, 25 % G, 25 % T, x 100 %
GN: 58 % A, 5 % C, 0 % G, 37 % T, x 100 %
E1: 25 % A, 25 % C, 25 % G, 25 % T, x 100 %
E2: 25 % A, 25 % C, 25 % G, 25 % T, x 100 %
-1: 5 % A, 45 % C, 5 % G, 45 % T, x 100 %
Ctarge: 0 % A, 100 % C, 0 % G, 0 % T, x 100 %
+1: 25 % A, 25 % C, 25 % G, 25 % T, x 100 %
3' end

Genome scan, request 36101
dsn3PLS-DYW_w_accessory_CDS

Top 40 hits

No.	Accession	Taxon	Organelle	Search result location	Scores/sequence ?	Total (max. 1029)
1	lcl|NZ_CP010816.1_cds_WP_000469031.1_751 [gene=ybhR] [locus_tag=SR36_RS03890] [protein=ABC transporter permease] [protein_id=WP_000469031.1] [location=complement(778159..779265)] [gbkey=CDS]			82..98	83 74 82 74 25 86 82 74 72 74 25 58 25 25 45 100 25<br>T T A C C C G T G C T A A T T C A	1029
2	lcl|NZ_CP010816.1_cds_WP_000188262.1_4162 [gene=fecA] [locus_tag=SR36_RS21700] [protein=TonB-dependent Fe(3+) dicitrate receptor FecA] [protein_id=WP_000188262.1] [location=complement(4396915..4399239)] [gbkey=CDS]			743..759	83 74 82 74 25 86 82 74 72 74 25 58 25 25 45 100 25<br>T T A C C C G T G C C A T T C C G	1029
3	lcl|NZ_CP010816.1_cds_WP_000570668.1_3751 [gene=typA] [locus_tag=SR36_RS19480] [protein=ribosome-dependent GTPase TypA] [protein_id=WP_000570668.1] [location=3934270..3936093] [gbkey=CDS]			complement(249..265)	83 74 82 74 25 86 82 74 20 74 25 58 25 25 45 100 25<br>T T A C A C G T T C A A C T T C A	977
4	lcl|NZ_CP010816.1_cds_WP_000940019.1_2407 [gene=trmJ] [locus_tag=SR36_RS12400] [protein=tRNA (cytosine(32)/uridine(32)-2'-O)-methyltransferase TrmJ] [protein_id=WP_000940019.1] [location=complement(2497553..2498293)] [gbkey=CDS]			647..663	83 74 82 74 25 86 82 74 72 74 25 5 25 25 45 100 25<br>T T A C C C G T G C G C G C C C G	976
5	lcl|NZ_CP010816.1_cds_WP_001321683.1_3488 [gene=radC] [locus_tag=SR36_RS18065] [protein=DNA repair protein RadC] [protein_id=WP_001321683.1] [location=complement(3647846..3648514)] [gbkey=CDS]			598..614	83 74 82 74 25 86 82 74 72 74 25 5 25 25 45 100 25<br>T T A C G C G T G C T C G A C C A	976
6	lcl|NZ_CP010816.1_cds_WP_000982447.1_2100 [gene=ccmF] [locus_tag=SR36_RS10815] [protein=cytochrome c-type biogenesis heme lyase CcmF] [protein_id=WP_000982447.1] [location=complement(2155776..2157719)] [gbkey=CDS]			complement(1246..1262)	83 74 82 74 25 86 82 20 72 74 25 58 25 25 45 100 25<br>T T A C G C G G G C G A T C C C G	975
7	lcl|NZ_CP010816.1_cds_WP_000717694.1_2428 [gene=glnB] [locus_tag=SR36_RS12505] [protein=nitrogen regulatory protein P-II] [protein_id=WP_000717694.1] [location=complement(2522040..2522378)] [gbkey=CDS]			complement(282..298)	83 20 82 74 25 86 82 74 72 74 25 58 25 25 45 100 25<br>T G A C C C G T G C C A C G T C A	975
8	lcl|NZ_CP010816.1_cds_WP_000656029.1_2678 [gene=yqeF] [locus_tag=SR36_RS13870] [protein=acetyl-CoA C-acetyltransferase] [protein_id=WP_000656029.1] [location=complement(2793304..2794485)] [gbkey=CDS]			805..821	83 74 82 74 25 86 82 74 72 74 25 0 25 25 45 100 25<br>T T A C C C G T G C T G G C C C G	971
9	lcl|NZ_CP010816.1_cds_WP_001296867.1_2259 [gene=yfdE] [locus_tag=SR36_RS11620] [protein=CoA:oxalate CoA-transferase] [protein_id=WP_001296867.1] [location=complement(2335076..2336221)] [gbkey=CDS]			844..860	83 74 82 74 25 86 82 74 8 74 25 58 25 25 45 100 25<br>T T A C G C G T A C A A A A C C A	965
10	lcl|NZ_CP010816.1_cds_WP_000125638.1_1580 [gene=malX] [locus_tag=SR36_RS08140] [protein=PTS maltose transporter subunit IICB] [protein_id=WP_000125638.1] [location=1613716..1615308] [gbkey=CDS]			complement(261..277)	83 74 15 74 25 86 82 74 72 74 25 58 25 25 45 100 25<br>T T T C G C G T G C C A G A C C C	962
11	lcl|NZ_CP010816.1_cds_WP_000781888.1_1720 [gene=nudG] [locus_tag=SR36_RS08860] [protein=CTP pyrophosphohydrolase] [protein_id=WP_000781888.1] [location=1755031..1755438] [gbkey=CDS]			52..68	83 74 82 74 25 86 82 6 72 74 25 58 25 25 45 100 25<br>T T A C T C G C G C A A C G C C C	961
12	lcl|NZ_CP010816.1_cds_WP_000083017.1_2462 [gene=pat] [locus_tag=SR36_RS12695] [protein=protein lysine acetyltransferase] [protein_id=WP_000083017.1] [location=2555718..2558378] [gbkey=CDS]			2387..2403	83 6 82 74 25 86 82 74 72 74 25 58 25 25 45 100 25<br>T C A C A C G T G C G A T C T C C	961
13	lcl|NZ_CP010816.1_cds_WP_001253612.1_3088 [gene=yhdP] [locus_tag=SR36_RS15960] [protein=AsmA2 domain-containing protein YhdP] [protein_id=WP_001253612.1] [location=complement(3222779..3226579)] [gbkey=CDS]			863..879	83 74 82 74 25 86 82 74 72 6 25 58 25 25 45 100 25<br>T T A C G C G T G A A A A T C C G	961
14	lcl|NZ_CP010816.1_cds_WP_001181324.1_4113 [gene=treR] [locus_tag=SR36_RS21435] [protein=trehalose operon repressor TreR] [protein_id=WP_001181324.1] [location=complement(4347106..4348053)] [gbkey=CDS]			complement(24..40)	83 74 82 6 25 86 82 74 72 74 25 58 25 25 45 100 25<br>T T A A G C G T G C G A T G T C T	961
15	lcl|NZ_CP010816.1_cds_WP_001299799.1_4268 [gene=yjjU] [locus_tag=SR36_RS22260] [protein=patatin family protein] [protein_id=WP_001299799.1] [location=4499951..4501024] [gbkey=CDS]			228..244	83 74 82 74 25 86 82 6 72 74 25 58 25 25 45 100 25<br>T T A C G C G C G C A A A G T C A	961
16	lcl|NZ_CP010816.1_cds_WP_000010149.1_1788 [gene=rlmA] [locus_tag=SR36_RS09215] [protein=23S rRNA (guanine(745)-N(1))-methyltransferase] [protein_id=WP_000010149.1] [location=complement(1821143..1821952)] [gbkey=CDS]			complement(486..502)	10 74 82 74 25 86 82 74 72 74 25 58 25 25 45 100 25<br>C T A C T C G T G C T A A T T C T	956
17	lcl|NZ_CP010816.1_cds_WP_000579833.1_3154 [gene=rplC] [locus_tag=SR36_RS16350] [protein=50S ribosomal protein L3] [protein_id=WP_000579833.1] [location=complement(3282632..3283261)] [gbkey=CDS]			complement(495..511)	83 74 82 74 25 86 82 74 20 74 25 37 25 25 45 100 25<br>T T A C A C G T T C G T T A C C C	956
18	lcl|NZ_CP010816.1_cds_WP_012767757.1_2796 [locus_tag=SR36_RS14455] [protein=polysaccharide biosynthesis/export family protein] [protein_id=WP_012767757.1] [location=2917092..2918768] [gbkey=CDS]			1610..1626	83 74 82 74 25 86 82 74 72 0 25 58 25 25 45 100 25<br>T T A C C C G T G G T A T T T C C	955
19	lcl|NZ_CP010816.1_cds_WP_000644741.1_3146 [gene=rpmC] [locus_tag=SR36_RS16310] [protein=50S ribosomal protein L29] [protein_id=WP_000644741.1] [location=complement(3278903..3279094)] [gbkey=CDS]			complement(144..160)	83 0 82 74 25 86 82 74 72 74 25 58 25 25 45 100 25<br>T A A C G C G T G C G A C A T C G	955
20	lcl|NZ_CP010816.1_cds_WP_001196486.1_3382 [gene=dppD] [locus_tag=SR36_RS17520] [protein=dipeptide ABC transporter ATP-binding protein] [protein_id=WP_001196486.1] [location=complement(3534184..3535167)] [gbkey=CDS]			306..322	83 0 82 74 25 86 82 74 72 74 25 58 25 25 45 100 25<br>T A A C C C G T G C T A C A C C G	955
21	lcl|NZ_CP010816.1_cds_WP_000098413.1_392 [gene=ampG] [locus_tag=SR36_RS02010] [protein=muropeptide MFS transporter AmpG] [protein_id=WP_000098413.1] [location=complement(419754..421229)] [gbkey=CDS]			complement(1218..1234)	83 74 82 74 25 86 82 74 72 20 25 37 25 25 45 100 25<br>T T A C T C G T G T A T A T T C A	954
22	lcl|NZ_CP010816.1_cds_WP_000107384.1_966 [gene=hyaB] [locus_tag=SR36_RS04980] [protein=Ni/Fe-hydrogenase large subunit] [protein_id=WP_000107384.1] [location=1007508..1009301] [gbkey=CDS]			1719..1735	83 20 82 74 25 86 82 74 72 74 25 37 25 25 45 100 25<br>T G A C C C G T G C C T C G C C T	954
23	lcl|NZ_CP010816.1_cds_WP_085947771.1_1022 [locus_tag=SR36_RS05265] [protein=IS3-like element IS3 family transposase] [exception=ribosomal slippage] [protein_id=WP_085947771.1] [location=complement(join(1067723..1068624,1068624..1068885))] [gbkey=CDS]			complement(409..425)	83 20 82 74 25 86 82 74 72 74 25 37 25 25 45 100 25<br>T G A C G C G T G C T T A T C C T	954
24	lcl|NZ_CP010816.1_cds_WP_085947771.1_1367 [locus_tag=SR36_RS07055] [protein=IS3-like element IS3 family transposase] [exception=ribosomal slippage] [protein_id=WP_085947771.1] [location=join(1395819..1396080,1396080..1396981)] [gbkey=CDS]			complement(409..425)	83 20 82 74 25 86 82 74 72 74 25 37 25 25 45 100 25<br>T G A C G C G T G C T T A T C C T	954
25	lcl|NZ_CP010816.1_cds_WP_085947598.1_1517 [locus_tag=SR36_RS07820] [protein=IS3-like element IS3 family transposase] [exception=ribosomal slippage] [protein_id=WP_085947598.1] [location=join(1556161..1556422,1556422..1557323)] [gbkey=CDS]			complement(409..425)	83 20 82 74 25 86 82 74 72 74 25 37 25 25 45 100 25<br>T G A C G C G T G C T T A T C C T	954
26	lcl|NZ_CP010816.1_cds_WP_085947598.1_2681 [locus_tag=SR36_RS13890] [protein=IS3-like element IS3 family transposase] [exception=ribosomal slippage] [protein_id=WP_085947598.1] [location=join(2797046..2797307,2797307..2798208)] [gbkey=CDS]			complement(409..425)	83 20 82 74 25 86 82 74 72 74 25 37 25 25 45 100 25<br>T G A C G C G T G C T T A T C C T	954
27	lcl|NZ_CP010816.1_cds_WP_000196054.1_3423 [gene=lyxK] [locus_tag=SR36_RS17740] [protein=L-xylulokinase] [protein_id=WP_000196054.1] [location=3578401..3579897] [gbkey=CDS]			573..589	83 20 82 74 25 86 82 74 72 74 25 37 25 25 45 100 25<br>T G A C C C G T G C C T C A C C G	954
28	lcl|NZ_CP010816.1_cds_SR36_RS19990_3848 [gene=btuB] [locus_tag=SR36_RS19990] [protein=TonB-dependent vitamin B12 receptor BtuB] [pseudo=true] [location=4041105..4042949] [gbkey=CDS]			complement(543..559)	83 74 82 74 25 86 82 74 72 20 25 37 25 25 45 100 25<br>T T A C C C G T G T C T T A T C C	954
29	lcl|NZ_CP010816.1_cds_WP_001226602.1_3657 [gene=rffG] [locus_tag=SR36_RS18965] [protein=dTDP-glucose 4,6-dehydratase] [protein_id=WP_001226602.1] [location=3831927..3832994] [gbkey=CDS]			complement(192..208)	7 74 82 74 25 86 82 74 72 74 25 58 25 25 45 100 25<br>A T A C G C G T G C C A G T T C T	953
30	lcl|NZ_CP010816.1_cds_WP_000106833.1_1688 [gene=hxpB] [locus_tag=SR36_RS08695] [protein=hexitol phosphatase HxpB] [protein_id=WP_000106833.1] [location=1722958..1723626] [gbkey=CDS]			239..255	83 74 0 74 25 86 82 74 72 74 25 58 25 25 45 100 25<br>T T G C C C G T G C C A T T T C A	947
31	lcl|NZ_CP010816.1_cds_WP_000050798.1_2093 [gene=yejK] [locus_tag=SR36_RS10770] [protein=nucleoid-associated protein YejK] [protein_id=WP_000050798.1] [location=complement(2146954..2147961)] [gbkey=CDS]			complement(741..757)	83 74 82 74 25 86 0 74 72 74 25 58 25 25 45 100 25<br>T T A C G C C T G C C A G C T C T	947
32	lcl|NZ_CP010816.1_cds_WP_000979881.1_3064 [gene=nanQ] [locus_tag=SR36_RS15840] [protein=N-acetylneuraminate anomerase] [protein_id=WP_000979881.1] [location=complement(3199342..3199806)] [gbkey=CDS]			complement(185..201)	83 74 0 74 25 86 82 74 72 74 25 58 25 25 45 100 25<br>T T G C T C G T G C A A T T C C G	947
33	lcl|NZ_CP010816.1_cds_WP_001297265.1_3249 [gene=gntX] [locus_tag=SR36_RS16835] [protein=DNA utilization protein GntX] [protein_id=WP_001297265.1] [location=3374408..3375091] [gbkey=CDS]			547..563	83 74 0 74 25 86 82 74 72 74 25 58 25 25 45 100 25<br>T T G C C C G T G C A A G G T C G	947
34	lcl|NZ_CP010816.1_cds_WP_000448135.1_3261 [gene=glpD] [locus_tag=SR36_RS16895] [protein=glycerol-3-phosphate dehydrogenase] [protein_id=WP_000448135.1] [location=3391475..3392980] [gbkey=CDS]			506..522	0 74 82 74 25 86 82 74 72 74 25 58 25 25 45 100 25<br>G T A C T C G T G C T A C T T C T	946
35	lcl|NZ_CP010816.1_cds_WP_001226164.1_229 [gene=dinB] [locus_tag=SR36_RS01190] [protein=DNA polymerase IV] [protein_id=WP_001226164.1] [location=255464..256519] [gbkey=CDS]			complement(420..436)	83 74 82 74 25 2 82 74 72 74 25 58 25 25 45 100 25<br>T T A C G G G T G C C A C G C C C	945
36	lcl|NZ_CP010816.1_cds_WP_001020381.1_2693 [gene=yqeB] [locus_tag=SR36_RS13955] [protein=selenium-dependent molybdenum cofactor biosynthesis protein YqeB] [protein_id=WP_001020381.1] [location=complement(2812641..2814266)] [gbkey=CDS]			complement(1134..1150)	83 74 82 74 25 2 82 74 72 74 25 58 25 25 45 100 25<br>T T A C T G G T G C C A T A T C T	945
37	lcl|NZ_CP010816.1_cds_WP_000836067.1_1538 [gene=rspB] [locus_tag=SR36_RS07930] [protein=Zn-dependent oxidoreductase] [protein_id=WP_000836067.1] [location=complement(1567258..1568277)] [gbkey=CDS]			complement(72..88)	83 74 82 74 25 86 82 74 8 74 25 37 25 25 45 100 25<br>T T A C T C G T A C T T C A C C C	944
38	lcl|NZ_CP010816.1_cds_WP_000255051.1_2055 [gene=mglA] [locus_tag=SR36_RS10570] [protein=galactose/methyl galactoside ABC transporter ATP-binding protein MglA] [protein_id=WP_000255051.1] [location=complement(2101838..2103358)] [gbkey=CDS]			complement(1014..1030)	83 74 82 74 25 0 82 74 72 74 25 58 25 25 45 100 25<br>T T A C C A G T G C A A A T C C A	943
39	lcl|NZ_CP010816.1_cds_WP_000015497.1_2565 [gene=ygbA] [locus_tag=SR36_RS13280] [protein=nitrous oxide-stimulated promoter family protein] [protein_id=WP_000015497.1] [location=complement(2665585..2665938)] [gbkey=CDS]			271..287	83 74 82 6 25 86 82 74 72 74 25 37 25 25 45 100 25<br>T T A A C C G T G C G T C A T C T	940
40	lcl|NZ_CP010816.1_cds_WP_000422149.1_2946 [gene=yqjA] [locus_tag=SR36_RS15240] [protein=DedA family general envelope maintenance protein YqjA] [protein_id=WP_000422149.1] [location=3083237..3083899] [gbkey=CDS]			316..332	83 74 82 74 25 86 82 6 72 74 25 37 25 25 45 100 25<br>T T A C C C G C G C A T T A T C A	940

Back to input page

CCCCGAGAACCTGCGTGTACT
Version: 3.12.0 using NCBI BLAST 2.2.26+
Lenz *et al.* 2010; Lenz and Knoop 2013; Lenz *et al.* 2018

Figure S11. Weight matrix used for prediction of dsn3PLS-DYW cytidine targets within the *E.coli* CDS by the TargetScan tool of PREPACT and the scanning output with the 40 best predicted targets. (Top) Each column corresponds to one nucleotide position and is labelled either (i) with type and fifth and last amino acid of the corresponding PPR or (ii) with the position relative to the editing site (EdS). Nucleotide distributions were set according to the published PPR-RNA binding code. Positional weighs are shown below. (Bottom) Best 40 predicted targets with the position in the genome and the matrix score (maximum 1000). Matches are colored in green and mismatches in red and given are the percentages of the match. The red stars indicate the two sites that have been detected among the 34 observed off-targets (Table 2).

	from	to	Strand	Sample1	Sample2	Sample3	Sample4	Sample5	Sample6	Sample7	Sample8	Sample9	Sample10	Sample11	Sample12	Sample13	Sample14
★	778159	779265	-	2.312	0.993	1.163	1.196	1.103	1.143	0.951	0.991	1.223	0.936	1.113	0.934	0.968	1.069
	4396915	4399239	-	0.086	0.014	0.012	0.036	0.017	0.019	0.013	0.019	0.021	0.020	0.017	0.022	0.020	0.021
	3934270	3936093	-	4.985	7.162	4.001	4.408	6.139	8.534	7.698	6.791	6.676	5.630	5.004	4.848	4.880	3.700
	2497553	2498293	-	0.155	0.064	0.038	0.110	0.097	0.049	0.053	0.073	0.106	0.072	0.083	0.027	0.024	0.036
	3647846	3648514	-	0.264	0.030	0.023	0.086	0.027	0.031	0.023	0.045	0.048	0.030	0.031	0.018	0.028	0.028
	2155776	2157719	+	0.063	0.003	0.003	0.015	0.006	0.006	0.003	0.007	0.009	0.008	0.006	0.003	0.003	0.003
	2522040	2522378	+	0.504	0.166	0.116	0.530	0.288	0.347	0.161	0.228	0.350	0.233	0.334	0.072	0.067	0.095
	2793304	2794485	-	0.171	0.016	0.009	0.043	0.014	0.022	0.013	0.030	0.028	0.018	0.016	0.009	0.010	0.014
	2335076	2336221	-	0.490	0.044	0.029	0.179	0.031	0.082	0.043	0.052	0.076	0.046	0.041	0.006	0.010	0.022
	1613716	1615308	-	2.278	1.407	1.072	1.748	1.660	1.487	1.624	1.465	1.726	1.809	1.722	0.612	0.674	0.872
	1755031	1755438	+	0.390	0.249	0.209	0.316	0.236	0.201	0.205	0.206	0.290	0.231	0.245	0.150	0.158	0.220
	2555718	2558378	+	0.605	0.430	0.516	0.310	0.341	0.623	0.428	0.431	0.322	0.466	0.246	2.255	1.976	1.334
	3222779	3226579	-	0.043	0.008	0.006	0.020	0.011	0.006	0.005	0.012	0.013	0.011	0.010	0.009	0.010	0.009
	4347106	4348053	+	0.140	0.026	0.014	0.070	0.026	0.042	0.018	0.039	0.047	0.034	0.030	0.029	0.026	0.035
	4499951	4501024	+	0.750	0.845	0.638	0.713	0.666	0.774	0.726	0.654	0.634	0.714	0.621	1.936	1.751	1.288
★	1821143	1821952	+	0.369	0.163	0.071	0.225	0.130	0.187	0.128	0.146	0.175	0.169	0.181	0.068	0.074	0.092
	3282632	3283261	+	0.073	0.010	0.009	0.011	0.010	0.015	0.017	0.020	0.013	0.013	0.010	0.032	0.011	0.014
	2917092	2918768	+	0.247	0.086	0.061	0.145	0.062	0.109	0.090	0.106	0.125	0.092	0.082	0.044	0.048	0.050
	3278903	3279094	+	0.109	0.034	0.027	0.057	0.040	0.034	0.041	0.054	0.073	0.072	0.043	0.057	0.031	0.039
	3534184	3535167	-	0.982	0.063	0.042	0.384	0.083	0.110	0.054	0.119	0.164	0.115	0.081	0.005	0.017	0.032
	419754	421229	+	0.117	0.044	0.039	0.083	0.059	0.049	0.041	0.043	0.062	0.045	0.055	0.057	0.055	0.060
	1007508	1009301	+	0.079	0.023	0.015	0.034	0.025	0.029	0.023	0.029	0.026	0.020	0.016	0.095	0.064	0.028
	1067723	1068624	+	0.648	0.157	0.105	0.302	0.172	0.165	0.132	0.168	0.258	0.197	0.182	0.054	0.071	0.100
	1395819	1396981	-	0.313	0.107	0.106	0.175	0.106	0.155	0.135	0.122	0.143	0.130	0.096	0.169	0.148	0.125
	1556161	1557323	-	0.382	0.105	0.099	0.236	0.105	0.151	0.122	0.134	0.143	0.131	0.097	0.148	0.144	0.107
	2797046	2798208	-	0.305	0.106	0.091	0.187	0.109	0.146	0.139	0.122	0.145	0.136	0.097	0.154	0.138	0.110
	3578401	3579897	+	0.161	0.026	0.019	0.070	0.032	0.034	0.030	0.039	0.051	0.031	0.029	0.014	0.017	0.020
	4041105	4042949	-	1.245	0.331	0.256	0.415	0.262	0.494	0.313	0.413	0.475	0.374	0.317	0.281	0.315	0.308
	3831927	3832994	-	1.792	0.762	0.374	0.346	0.321	0.953	0.612	0.605	0.475	0.523	0.281	1.260	1.405	0.779
	1722958	1723626	+	1.110	1.087	1.257	0.971	0.835	1.330	1.371	1.181	1.124	1.138	0.833	1.262	1.274	1.239
	2146954	2147961	+	0.100	0.026	0.017	0.059	0.032	0.023	0.026	0.029	0.059	0.054	0.039	0.007	0.007	0.012
	3199342	3199806	+	2.274	0.472	0.402	0.913	0.416	0.469	0.364	0.580	0.755	0.595	0.556	0.291	0.316	0.466
	3374408	3375091	+	0.792	0.971	2.557	0.969	0.945	1.177	1.552	0.972	1.138	1.390	0.877	1.720	1.697	1.469
	3391475	3392980	+	0.699	0.653	0.170	1.572	0.396	1.385	0.649	0.605	1.049	1.410	0.365	2.489	1.644	0.869
	255464	256519	-	0.661	0.444	0.247	0.569	0.439	0.395	0.368	0.400	0.663	0.466	0.377	0.160	0.129	0.223
	2812641	2814266	+	0.055	0.012	0.008	0.029	0.011	0.011	0.009	0.008	0.018	0.014	0.013	0.007	0.005	0.008
	1567258	1568277	+	0.296	0.071	0.085	0.161	0.064	0.108	0.074	0.119	0.137	0.109	0.114	0.032	0.033	0.050
	2101838	2103358	+	0.091	0.016	0.014	0.063	0.013	0.019	0.016	0.014	0.027	0.021	0.019	0.003	0.002	0.007
	2665585	2665938	-	0.073	0.024	0.018	0.049	0.021	0.030	0.030	0.019	0.029	0.025	0.025	0.091	0.105	0.045
	3083237	3083899	+	2.104	0.881	0.939	1.578	1.832	1.018	1.206	1.497	2.233	1.107	1.637	0.987	0.991	1.246

Figure S12. Gene expression levels of genes identified as theoretical off-targets by PREPACT3. The expression levels were quantified using samtools (version 1.13) to count the total mapped transcripts. The total reads were normalized to reads per nucleotide (rpn). Relative expression was calculated by dividing the rpn of each gene by the mean rpn of the housekeeping genes cysG and heaT for each sample. The shade of the red background indicates the level of expression, the darker the background, the higher the expression. The red stars indicate the two sites that have been detected among the 34 observed off-targets (Table 2). One of the observed off-target (1821143-1821952) has a relatively low level of expression.
